# Reemergence of Strongyloidiasis, Northern Italy

**DOI:** 10.3201/eid1509.090191

**Published:** 2009-09

**Authors:** Fabrizio F. Abrescia, Alessandra Falda, Giacomo Caramaschi, Alfredo Scalzini, Federico Gobbi, Andrea Angheben, Maria Gobbo, Renzo Schiavon, Pierangelo Rovere, Zeno Bisoffi

**Affiliations:** Sacro Cuore Hospital, Verona, Italy (F.F. Abrescia, F. Gobbi, A. Angheben, M. Gobbo, Z. Bisoffi); C. Poma Hospital, Mantova, Italy (A. Falda, G. Caramaschi, A. Scalzini); Mater Salutis Hospital, Legnago, Verona (R. Schiavon, P. Rovere).

**Keywords:** Strongyloidiasis, eosinphilia, elderly, helminth, parasites, letter

**To the Editor:** Strongyloidiasis is a helminth infection caused by *Strongyloides stercoralis,* a nematode ubiquitous in tropical and subtropical countries and occasionally reported in temperate countries, including Italy (*1*). Sources of infection are filariform strongyloid larvae present in soil contaminated by infected feces; the larvae penetrate through the skin of a human host. After the first life cycle, a process of autoinfection begins, which persists indefinitely in the host if the infection is not effectively treated. The infection can remain totally asymptomatic for many years or forever or cause cutaneous (itching and rash), abdominal (epigastric pain, pseudoappendicitis, diarrhea), respiratory (cough, recurrent asthma), and systemic (weight loss, cachexia) symptoms that can be enervating. More importantly, when host immunity is impaired because of a concurrent disease or immunosuppressive therapy (including corticosteroids, sometimes used to treat symptoms of the unrecognized infection or the concurrent eosinophilia), disseminated strongyloidiasis may occur (*2*–*4*), causing a massive and almost invariably fatal invasion of virtually all organs and tissues by filariform larvae and even adult worms (Figure), often combined with bacterial superinfection. This complication is believed to be rare but is probably underestimated because of the extreme variability of the clinical presentation.

**Figure:**
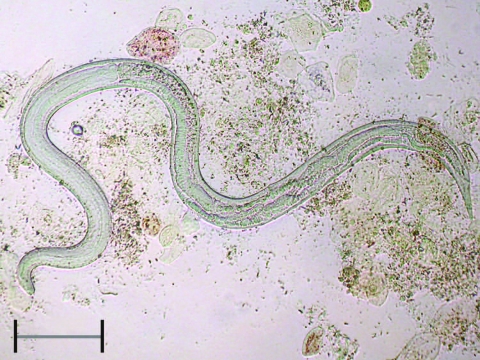
Adult female of *Strongyloides stercoralis* collected in bronchial fluid of a patient with disseminated disease. Scale bar = 400 µm.

Although strongyloidiasis can be suspected in the presence of symptoms or eosinophilia (which is frequent but not mandatory), the low sensitivity of direct diagnostic methods often lets the disease go unrecognized (*5*–*7*). By far the most sensitive diagnostic tools are serologic tests: sensitivity and specificity of indirect fluorescent antibody test (IFAT) (in-house produced IFAT) are 97.4% and 97.9%, respectively, at a dilution >1/20, and 70.5% and 99.8% at a dilution >1/80 (*6*). A suspected case is defined by a positive antibody titer >20 (IFAT); a case is confirmed by a positive direct test result (culture in agar being the most sensitive direct technique) or by a positive antibody titer >80 (*6*). Despite some anecdotal reports on the presence of strongyloidiasis in Italy (*1*,*6*), reliable information about the real prevalence of the infection is lacking. After seeing several patients affected by the disease, 1 of whom died because of dissemination **(**Z. Bisoffi, unpub. data), we decided to carry out a preliminary rapid assessment of the extent of the problem in elderly patients with eosinophilia.

During a 4-month period, from February through May 2008, every patient born in 1940 or earlier who came to the clinical laboratories of 2 contiguous health districts in northern Italy (Mantova, Lombardy Region, and Legnago, Veneto Region) for a diagnostic blood test (hematocrit and leukocyte count/formula) for whatever reason and having a eosinophil count >500 cells/μL was asked to join the study. This study was the pilot phase of a larger, multicentered study, which obtained formal approval from the Ethical Committee of Sacro Cuore Hospital of Negrar, Verona. Informed consent was required of each patient. Of the 132 patients eligible for inclusion (mean age 76.4 years, range 68–90 years, male:female ratio 1.6), none refused to give informed consent. Serum specimens were subjected to the IFAT for *S. stercoralis* at the Sacro Cuore Hospital Centre for Tropical Diseases.

Unexpectedly, we found that 37 (28%) of 132 patients were positive, with titers ranging between 20 and >320 (and >80 in most cases). However, caution should be exercised in interpreting the results because the patients may not be representative of the general population. Moreover, our results are based on an indirect (although highly sensitive and specific) test. Because the reported cases involve only a few patients every year (of whom some are anecdotally reported as dying from the infection, usually unpublished), we suspect that most strongyloidiasis cases remain undetected.

If relevant transmission still exists in the area, it is unknown but is unlikely because of the improvement of hygienic conditions in the past 5 decades. Reports of the infection in children or young adults with no travel history outside Italy are lacking. Strongyloidiasis in the elderly is therefore most likely to result from an infection that occurred much earlier in life, either in infancy or at a young age, while walking or working barefoot in agricultural fields. The long persistence is the consequence of the autoinfection cycle typical of this parasite as described above. The result is an important and unrecognized public health problem affecting the geriatric population of northern Italy. These preliminary results confirm the need for the already planned, multicentered study involving a larger sample and a wider geographic area.
